# Experimental Evolution as an Underutilized Tool for Studying Beneficial Animal–Microbe Interactions

**DOI:** 10.3389/fmicb.2016.01444

**Published:** 2016-09-13

**Authors:** Kim L. Hoang, Levi T. Morran, Nicole M. Gerardo

**Affiliations:** Department of Biology, O. Wayne Rollins Research Center, Emory UniversityAtlanta, GA, USA

**Keywords:** host–microbe interactions, mutualisms, novel symbiosis, experimental evolution, animal models

## Abstract

Microorganisms play a significant role in the evolution and functioning of the eukaryotes with which they interact. Much of our understanding of beneficial host–microbe interactions stems from studying already established associations; we often infer the genotypic and environmental conditions that led to the existing host–microbe relationships. However, several outstanding questions remain, including understanding how host and microbial (internal) traits, and ecological and evolutionary (external) processes, influence the origin of beneficial host–microbe associations. Experimental evolution has helped address a range of evolutionary and ecological questions across different model systems; however, it has been greatly underutilized as a tool to study beneficial host–microbe associations. In this review, we suggest ways in which experimental evolution can further our understanding of the proximate and ultimate mechanisms shaping mutualistic interactions between eukaryotic hosts and microbes. By tracking beneficial interactions under defined conditions or evolving novel associations among hosts and microbes with little prior evolutionary interaction, we can link specific genotypes to phenotypes that can be directly measured. Moreover, this approach will help address existing puzzles in beneficial symbiosis research: how symbioses evolve, how symbioses are maintained, and how both host and microbe influence their partner’s evolutionary trajectories. By bridging theoretical predictions and empirical tests, experimental evolution provides us with another approach to test hypotheses regarding the evolution of beneficial host–microbe associations.

## Introduction

Microorganisms inhabit hosts from all branches of life, from bacteria (Bondy-Denomy and Davidson, 2014) to plants (Heijden et al., 2015) and animals (Klepzig et al., 2009; Delsuc et al., 2014), including humans (Cho and Blaser, 2012; Eloe-Fadrosh and Rasko, 2013). Associations with microbes play a pivotal role in the evolution and functioning of possibly all eukaryotes (Eloe-Fadrosh and Rasko, 2013; McFall-Ngai et al., 2013; Douglas, 2014; Rosenberg and Zilber-rosenberg, 2016). Host–microbe interactions can lead to a range of consequences for both the hosts and the microbes. These consequences vary along a continuum of parasitism to mutualism, a spectrum that represents the fitness costs and benefits for both the hosts and the microbes in association (Ewald, 1987; Gerardo, 2015). Host–microbe dynamics may not be static under all contexts (e.g., biotic and abiotic, temporal and spatial), and shifts along the continuum can occur if the net fitness benefit obtained for one partner (particularly the microbe) is higher than previous forms of interactions (Sachs and Simms, 2006).

Numerous microbes confer major benefits to their hosts. These benefits include nutrient provisioning and protection from enemies. Microbes also have essential roles in the development, functioning, and behavior of hosts. Association with microbes allows many hosts to inhabit niches that they normally would not be able to inhabit (McFall-Ngai et al., 2013; Douglas, 2014). Recognition of the importance of microbes in everyday life has heightened interests in understanding how beneficial host–microbe associations evolve, how the partners impact one another, and what are both the proximate and ultimate mechanisms behind these interactions. Important insights have resulted from studies of microbial symbiosis (here, defined as long-term host–microbe associations; Wilson et al., 2010; Sachs et al., 2011; Nyholm and Graf, 2012); however, there are still many questions that remain, particularly as to how beneficial associations are initiated between hosts and microbes with little prior interaction or with few benefits exchanged between them initially. Many of the conclusions we have drawn to date come from studies of existing relationships, often after an evolutionarily long period of coevolution between the partners. Factors that maintain associations now may not be the same as those that were present during the initial stages of the association, and the consequences (e.g., fitness costs and benefits, genomic modifications) have mostly been inferred from extant model systems. Here, we propose extending the use of experimental evolution as a way to fill in current knowledge gaps in beneficial host–microbe studies. This approach has helped to answer other fundamental evolutionary and ecological questions, but has still not been used to a great extent to study animal–microbe symbioses. The incorporation of experimental evolution into beneficial host–microbe interaction studies, a combination of approaches that is still in its infancy, will act to directly link proposed hypotheses to phenomena that can occur during the evolution of beneficial associations.

This review will be focused on highlighting the questions that remain unanswered in beneficial animal–microbe associations that are amenable to experimental evolution approaches. We then suggest ways in which experimental evolution can be used to address these questions and animal systems that may be exploited for these experiments moving forward. In this review, we define an interaction as “mutualistic” if a net fitness benefit is obtained by both host and microbe when they interact with each other compared to when they do not, and as a “beneficial association,” a more general term, when it is known that the host benefits (**Box 1** provides further details on the ambiguity of defining such associations).

**Box 1. Ambiguities in Defining “Mutualism”**

**Defining Host–Microbe Mutualisms**

Mutualism is most often defined as reciprocally beneficial interactions between species (Bergstrom et al., 2003; Bronstein, 2009). However, the term mutualism is sometimes used to describe associations conferring benefits to the partner of focus, which is usually the host (Ewald, 1987; Moran and Wernegreen, 2000; Perez-Brocal et al., 2011), or when a net benefit is obtained through partner exploitation (Herre et al., 1999). This is in part because it is often difficult to evaluate the consequences of an interaction for all partners involved. In relation to host–microbe symbioses, many studies have shown benefits conferred by microbes to hosts, but few have empirically demonstrated hosts conferring benefits to their symbiotic microbes (Garcia and Gerardo, 2014). Therefore, interactions that are referred to as mutualistic may not be true reciprocal mutualisms, in which both host and microbe fitness is enhanced.

Several considerations are worth noting when defining beneficial host–microbe interactions, making any consensus past a general definition of mutualism difficult to achieve. In facultative interactions, the association may be beneficial only under certain contexts (e.g., a specific environmental condition or presence of an enemy), and being associated with a microbial partner may actually be costly to the host when it does not provide benefits. Identifying the biotic and abiotic factors in context-dependent interactions is important in evaluating the role of genetics and the environment in the evolution and maintenance of mutualisms (Jones et al., 2015). For example, in their discussion of modes of beneficial behaviors, Sachs et al. (2004) introduced the idea of a parasite that can prevent the establishment of a more harmful parasite within a host. The less harmful parasite might be regarded as beneficial because the host benefits from the association with the less harmful parasite when the more harmful parasite is present. In the pea aphid (*Acyrthosiphon pisum*), Weldon et al. (2013) found that even though the bacterium *H. defensa* protects its aphid hosts from parasitoid wasps, the benefits are not conferred when the bacterium is no longer infected by the *A. pisum* secondary endosymbiont phage, and, in the absence of the phage, the host exhibits severe fitness costs when in partnership with the bacterium. Temporal context also should be taken into consideration: an interaction may not have been mutualistic in the initial stages of the association. For example, a microbe may become trapped within a host, where it does not grow as well as in the external environment. Over time, the microbe may evolve to utilize host resources, and eventually proliferate better than it would outside the host. In other words, the beneficial associations we see now may not have had a beneficial beginning.

**Mechanistic Similarities between Beneficial and Parasitic Symbionts**

Both beneficial and parasitic symbionts (microbes that form long-term association with hosts) must be able to overcome host defenses, to acquire and process host resources, and to compete with other microbes. Horizontal gene transfer facilitates successful infection of symbionts through acquisition of genes required for host interaction. These genes can be exchanged between beneficial and parasitic symbionts, suggesting that beneficial symbionts infect their hosts using mechanisms similar to parasitic ones (Hentschel et al., 2000; Perez-Brocal et al., 2011). Many obligate symbionts (those that cannot live in the absence of a host) also undergo genomic reduction as a result of adaptation to a relatively stable host environment (Ochman and Moran, 2001). While the mechanisms are similar, it is the net outcome of the interaction between molecular components of the microbe and host that determines where the interaction lies on the parasitism-mutualism continuum.

**Mutualism as an Arms Race**

Coevolution between some hosts and beneficial symbionts has been viewed as an arms race, not unlike between hosts and parasitic symbionts. Theories in host–microbe mutualism suggest that because beneficial symbionts are adapted to their hosts, they should be “evolutionarily static,” exhibiting slow rates of evolution and little genetic diversity to remain adapted to their hosts (i.e., any new variant of the symbiont is less likely to be suitable to a host; Law and Lewis, 1983). However, empirical studies have suggested similar evolutionary trajectories of beneficial and parasitic symbionts (such as rapid evolution and increased recombination), contradicting the previously proposed hypotheses of host–microbe mutualisms (Sachs et al., 2011). Thus, even in obligate symbioses where the fitnesses of the host and beneficial symbiont are the most closely aligned, the host must be able to respond to the rapidly evolving genome of its microbial partner. Because the microbial population is kept at a small size within hosts, genetic drift plays a large role in the genetic structure of the symbiont, leading to gene losses for which the host must compensate (Bennett and Moran, 2015).

## Beneficial Host–Microbe Interactions: Current Questions and Challenges

### The Initial Stages of Evolution

Past studies of host–microbe associations have focused on models that exhibit long-term, close–knit interactions, or at least where the host and microbe are known to have been in frequent contact with each other over evolutionary time (Douglas, 1998; Nyholm and McFall-Ngai, 2004). However, we have little evidence as to how these beneficial interactions evolve in the first place. One possibility is that these beneficial partnerships arise between hosts and microbes that have had little to no previous contact. For example, an animal might acquire a microbe from the environment that increases its fitness relative to its non-microbe harboring relatives, thus making this trait more prevalent in the population, particularly if eventually that microbe is transmitted vertically. Another possibility is that the initial association is commensal, parasitic, or predatory before selection for a beneficial interaction. Additionally, a situation may arise where this host–microbe pairing encounters a new environment, and it may be selectively beneficial for the microbe to now improve its host’s fitness. Discerning the origins of associations is challenging because current associations may be evolutionarily far removed from their pre-partnership ancestors.

Additionally, genetic and environmental conditions underlying already established symbioses may not be the same as when the host and microbe first came into contact. In terms of genetic conditions, this is likely particularly true for vertically transmitted symbioses due to the major genomic changes imposed by the host and microbe on each other as a result of their close–knit association (Bennett and Moran, 2015). Furthermore, studying existing associations does not allow us to directly assess the role that genetic variation had in establishing and maintaining long-term interactions. When there is a high rate of variation being generated through mutation, recombination, and gene flow during the initial interactions between host and microbe, beneficial associations may more likely evolve because there is more material for selection to act upon. However, it could also prevent the maintenance of a newly formed association because variation-generating mechanisms can disrupt allelic combinations that confer greater fitness (Fisher, 1930). Environmental conditions also likely have a large influence on the maintenance of associations. For example, even though the bacterium *Hamiltonella defensa* protects its pea aphid host from the parasitoid wasp, *Aphidius ervi*, the proportion of aphid hosts infected with the bacterium decreases in the absence of the wasp (Oliver et al., 2008), suggesting a cost in the absence of protection. Thus, fluctuation in wasp presence presumably leads to temporal shifts in the benefits and costs of association. More generally, the relative contributions of genetic and environmental factors and the degree to which these factors exert pressure on the maintenance of the first few generations of established symbioses remain unknown.

### Assessing Genomic Transitions Underlying Symbiosis Evolution

Past studies have shown that microbial lineages associated with a host often contain smaller genomes than their free-living counterparts. For example, in studies examining intracellular bacterial partners, or endosymbionts, genomic reduction is observed with increasing intensity of host association: facultative bacteria have smaller genomes than free-living bacteria, and obligate bacterial symbionts have smaller genomes than facultative bacterial symbionts (Toft and Andersson, 2010; McCutcheon and Moran, 2011). The evolution of endosymbionts, be they commensal, parasitic, or beneficial, has been proposed to involve free-living bacteria transitioning into a host-associated lifestyle. Once within the host, interactions with the host environment may render certain genes redundant (e.g., genes required for coping with external abiotic stressors, such as UV rays) in the endosymbiont. Combined with bottlenecks that occur when endosymbionts are passed on between hosts, genes necessary for free-living are lost. This leads to reductions in genome sizes and genetic variation in endosymbiont populations (Nilsson et al., 2005; Toft and Andersson, 2010; McCutcheon and Moran, 2011). One consequence is that interactions between host and endosymbiont can lead to complementarity of genomes, where host and endosymbiont produce resources their partner lacks (Wilson et al., 2010). For the symbiont, this could be the genes that they have lost, and for the host, the genes required for them to occupy a new niche. Furthermore, microbial genes can be integrated into the host genome through horizontal gene transfer, which has had an important role in eukaryotic evolution (Keeling and Palmer, 2008; McFall-Ngai et al., 2013; Rosenberg and Zilber-rosenberg, 2016).

These insights have all been gained from a comparative approach: genomes of symbionts are compared to those of free-living bacteria (e.g., Zheng et al., 2015) and genomes of hosts are compared to animals without intimate symbiotic relationships (e.g., Suen et al., 2011). Often, however, the free-living organisms are not closely related to the symbiotic organisms, so we cannot directly assess the genomic evolution underlying the transition from a free-living to a symbiotic lifestyle. In other words, we lack empirical evidence for inferences drawn from these genomic comparison studies. The challenge is that the ancestors are no longer present to perform direct tests of genomic changes resulting from symbiotic association. For example, a previous study sought to identify the genetic mechanisms involved in genomic reduction in bacteria by serially passaging single colonies over 200 times on supplemented media, effectively implementing strong bottlenecks and no horizontal gene transfer (Nilsson et al., 2005). Though the authors identified important aspects associated with genomic reduction (e.g., that it can happen in an evolutionarily short period of time), the experiments were done in the absence of any interaction with a host.

### The Role of the Host Immune System

The immune system is one of the most important lines of defense for the host; it acts as a way to differentiate between harmful and non-harmful microbes that colonize the host, whether they are environmentally acquired or passed down from the parent generation. Therefore, the immune system serves as a central component of the host that interacts intimately with its microbes. Studies of symbiotic interactions have shown that beneficial microbes have evolved ways to evade or alter host defenses, or have evolved from pathogenic ancestors and have retained their ability to evade or affect host immunity (Ruby, 2008; Nyholm and Graf, 2012). For example, when *Vibrio fischeri* bacteria colonize the light organ of their squid host, the bacteria actually dampen the level of host-produced nitric oxide, a compound involved in eukaryotic innate defense against pathogens (Davidson et al., 2004). Symbionts have also been shown to help regulate the development of normal immune responses and prime the host immune system to fight against pathogens, and the microbiota is required for proper host immune development and functioning in some organisms (Weiss et al., 2012; Yilmaz et al., 2014).

Despite the important role that the immune system has in mediating long-term host–microbe associations, we know little about the role it has in shaping the initial evolutionary stages of beneficial symbiosis. The innate immune system likely has an important role in the initial contact between host and microbe and evolution of a beneficial association due to its ability to shape and be shaped by microbes residing in the host (Nyholm and Graf, 2012; Weiss et al., 2012; Chu and Mazmanian, 2013). Theory suggests that host defenses can influence establishment of mutualism. For example, a heightened defense may impede the evolution of a symbiosis (Doebeli and Knowlton, 1998). However, most evolutionary theory regarding the establishment of symbiosis lacks direct empirical tests.

### The Maintenance of Mutualisms

Mutualisms are considered an evolutionary dilemma because individuals suffer costs to provide benefits to their partners; therefore, a long-standing question of interest has been to identify factors that stabilize or breakdown mutualisms. Mechanisms that promote shifting along the parasitism-mutualism spectrum and the genetic differences between parasites and symbionts are important factors that should be explored further (Sachs et al., 2011). Another related question is determining the contexts that give rise to cheating partners or endosymbiont reversion to free-living microbes (Sachs and Simms, 2006; Jones et al., 2015). Experimental evolution using tractable model systems provides a powerful way to examine these questions, as it has already been utilized to address related questions, such as the evolution of host-parasite interactions and novel microbe–microbe mutualisms.

## An Overview of Experimental Evolution

Experimental evolution refers to evolving populations under controlled conditions to study evolutionary processes (Garland and Rose, 2009; Kawecki et al., 2012). Precise conditions can be placed upon a population, which can then be tracked throughout its evolution. For instance, the relative strength of genetic drift can be manipulated by modifying population size, and selection can be altered by treatment effects or design. Control treatments can help distinguish between changes caused by the environment vs. evolutionary forces. Interestingly, replicate populations may also give rise to different adaptive genotypes that highlight the role of stochastic forces, like drift and mutation, in the evolutionary process. Richard Lenski’s long-term evolution experiment (LTEE), for example, has produced numerous examples of divergent adaptation found across 12 clonal *Escherichia coli* populations over 65,000 generations. While each *E. coli* population began as clones growing in identical environments, they eventually accumulated independent mutations that led to differential fitness gains on alternative sugars, as well as the emergence of one population that can utilize a carbon source, citrate, that *E. coli* normally cannot use (Travisano et al., 1995; Blount et al., 2008). Additionally, computer simulations can be a useful tool for experimental design in conjunction with experimental evolution, such as estimating the power of artificial selection experiments (Kessner and Novembre, 2015). Model systems that have been utilized in experimental evolution studies can be run for tens to thousands of generations, resulting in observation of evolution in real-time. Many organisms utilized for experimental evolution can be cryogenically preserved, allowing for direct comparisons of the ancestral populations against the evolved populations. The tractability of experimental evolution experiments makes them ideal for multispecies interaction studies, where environmental conditions are controlled to tease out the influences of biotic and abiotic factors on the evolution of the traits of interest.

Experimental evolution studies can be setup to test the effects of different variables on evolutionary trajectories. For example, in addition to exploring the adaptive mutations that arise across replicate populations, the LTEE has provided insight into fundamental evolutionary processes, such as diversification of clonal populations, the role of historical contingency in the evolution of novel traits, and the influence of mutation rates during adaptive evolution (Lenski et al., 1991; de Visser et al., 1999; Blount et al., 2008). Furthermore, a major benefit to experimental evolution is that it can be designed to test specific hypotheses. Biologists can control and isolate specific variables involved in a particular process, facilitating linkage between theory and models to empirical tests. It is important to note that it may be difficult to perfectly represent scenarios proposed by theories in laboratory experiments, which can lead to discrepancies between theories and empirical data (Desai, 2013). Computer simulations may provide a more controlled method to test predictions; however, organisms are more complex than simulations, so the utilization of living organisms in experimental evolution has the potential to reveal unknown biological phenomena, whereas it may not be possible to do so with simulations. For example, if we observed a particular phenotype in an evolved population, we can go back and identify the genes contributing to the particular trait, whereas we would require prior knowledge to perform simulations. More generally, experimental evolution serves as an intermediate between theories and natural populations. By evolving populations under controlled conditions, we can empirically test theoretical predictions, generate new data to parameterize models and simulations, and establish patterns to test in natural populations.

## Limitations

First, running experimental evolution under a controlled (often laboratory) environment may lead to simplification of the conditions found in nature, which are often more complex. More specifically, evolutionary processes may function differently in a natural environment where unidentified biotic and abiotic factors are in play, and multiple factors can act in synergy. For example, experimental coevolution of *Pseudomonas fluorescens* and its bacteriophage in rich medium led to directional selection of host-parasite dynamics, whereas coevolution of the same host-parasite pairing in soil microcosms resulted in frequency-dependent selection dynamics (Gómez and Buckling, 2011; Brockhurst and Koskella, 2013). A remedy to this dilemma may be the use of field experiments or mesocosms, which can more accurately represent the ecology of a particular system (Reznick et al., 1997; Ebert et al., 2002). However, one drawback may be less control over environmental conditions. While the laboratory may not completely encompass natural settings, a larger number of generations can be maintained in a controlled environment relative to those in the field, which is more subject to seasonal variability. Overall, experimental evolution is a useful tool in that it allows us to test what we predict as the most important factors involved in our study of interest and provides us with a starting point with which to test further predictions through field studies, simulations, quantitative and molecular genetics, and subsequent experimental evolution.

Second, in order to observe responses to selection in a set amount of time, extreme conditions are utilized to facilitate the evolutionary process, which may lead to phenotypic and genotypic patterns not observed in nature. For example, laboratory experiments tend to select for strong pleiotropic effects, while selection in nature often involve alleles with weak or no pleiotropy (Kawecki et al., 2012). Additionally, model organisms are restricted to those that have fast generation times if experiments begin with clonal populations (see **Box 2**) because otherwise mutations would not occur fast enough to provide the raw material for selection. This may be a problem because these organisms may not be those of interest for a particular question or may have particular peculiarities that limit generalization to other less rapidly reproducing organisms. This can be remedied, in part, by utilizing methods that increase genetic variation within populations (e.g., mutagenesis for standing genetic variation, or introducing defective DNA repair mechanisms). Despite limitations, experimental evolution studies have contributed significantly to existing areas of studies in ecology and evolution.

**Box 2. Approaches to Experimental Evolution**

**The Driving Mechanism: Natural Forces and Artificial Selection**

Classic experimental evolution studies involve studying how evolutionary processes shape populations. They seek to connect evolutionary forces to genotypic and phenotypic changes and to identify the molecular mechanisms involved. These changes are observed and measured before, during, and after specific conditions are set upon experimental populations. By contrast, artificial selection experiments shift the focus from evolutionary processes and toward selecting for certain phenotypes. The consequences of such selection can then be identified and measured after the desired traits are acquired. The most widely known example of artificial selection stems from selective breeding of animals and plants conducted by humans. Studies of domesticated plants and animals have also led to significant insight into the genetic changes resulting from adaptation of these organisms to human practices (Purugganan and Fuller, 2009; Andersson, 2012).

**The Model System: Single Species Evolution and Multiple-Species Interactions**

Fundamental evolutionary questions have been examined using populations of single species systems (Kawecki et al., 2012; Kassen, 2014). By implementing simple environmental conditions and limiting contact with unwanted organisms, this approach has addressed inquiries regarding the very core of evolutionary processes, such as the role of natural selection, gene flow, and genetic drift within and across populations. Multi-species studies have lent insights into processes that cannot be examined with single-species systems (e.g., Reznick et al., 1990; Hillesland and Stahl, 2010; Brockhurst and Koskella, 2013; Rouchet and Vorburger, 2014). For instance, coevolutionary dynamics between *P. fluorescens* and its phage were altered when a predator of the bacteria was introduced (Friman and Buckling, 2013). These experiments have provided insight into how predation, parasitism, and mutualism impact the evolution of the species involved. Multiple-species studies may be more ecologically representative because no species is completely isolated from others. Nonetheless, limiting interactions to a small number of species likely often fails to capture the true ecology in nature.

**The Starting Point: Clones and Standing Genetic Variation**

Studies solely using microbes tend to start with clonal populations. Because microbes have a short generation time, they can gain enough mutations within a short amount of time for evolutionary forces to act upon. Thus, mutations play a large role in generating genetic diversity in microbial studies. Independent accumulation of mutations can lead to divergence between clonal populations even under identical environments. Biologists can then track evolutionary trajectories taken by populations that begin identical to each other through comparison of ancestral and evolved populations using direct fitness tests. For model systems that have longer generation times (which are most eukaryotes), the starting populations tend to have standing genetic variation, which can be created through natural means (e.g., field collections) or genetic manipulations (e.g., mutagenesis). During the course of the experiment, variation can also be generated through recombination and outcrossing in populations that reproduce sexually. Although not as streamlined as clonal populations, these systems provide insight into the effects that evolutionary forces can have on populations where *de novo* mutation is not the only source of genetic diversity. Whether the populations are clones or contain standing genetic variation, there are usually multiple replicate populations in evolution experiments. This is to ascertain whether populations will converge on similar trajectories (when certain adaptations will arise predictably), or if the populations will diverge from each other (when rare changes are more important than common and predictable changes).

**The Environment: Laboratory and Field Experiments**

Many experimental evolution studies are run in a laboratory setting. This allows for more control and reproducibility of environmental conditions. The extent to which laboratory conditions represent natural conditions can vary across studies, and the importance of this representation can vary across questions. If a study is focused on broad evolutionary questions that can be applied across many organisms, environments that do not fully represent the natural setting may suffice, as more ecologically sound conditions can be added or modified in studies directed toward specific organisms. Field studies provide a more accurate representation of natural processes, but some conditions can be irreproducible or unidentified, making replications of experimental conditions difficult. Kawecki et al. (2012) suggests evolving populations under laboratory conditions (such as selecting for cold tolerance), and performing field experiments with the evolved populations to determine whether they are well-adapted to the natural environment (such as a cold environment).

**Evolution from Scratch: Synthetic Ecology and Digital Organisms**

Questions involving ecology and evolution have mainly involved studies of natural or existing populations and communities (Reznick et al., 1997; Scarborough et al., 2005; Zhen et al., 2012). Using information gained from these natural systems, we can implement another approach to generate hypotheses: creating synthetic or digital populations and communities with defined traits and observing how they evolve. Combined with advances in technology, these approaches can help us determine the evolutionary forces and mechanisms involved in the adaptation of natural populations throughout time.

Several experimental evolution studies have used constructed mutants as the starting population (Shou et al., 2007; Harcombe, 2010; Marchetti et al., 2010). By evolving populations carrying specific genes, we can identify the proximate mechanisms involved in the evolution of natural populations. Furthermore, the recently emerged field of synthetic ecology focuses on establishing communities composed of different microbial members to examine the consequences of species interaction, particularly for use in biotechnological developments. These experiments are generally composed of engineered mutants or microbes that are not naturally associated with each other (Escalante et al., 2015; Fredrickson, 2015).

Evolution has also been studied using digital organisms, which dwell and replicate inside of computers under user-selected settings [a type of agent-based model, with the most common platform being Avida (Ofria and Wilke, 2004)]. The requirements for evolution are simulated through digital equivalents, such as executable codes representing genomes, replication error probabilities representing mutation rates, and limited computer space and energy units for growth (Adami, 2006). Similarly, robots have also been used to study how communication evolves on the individual and colony levels (Floreano et al., 2007). Although there are limitations in using these digital platforms, they have provided support for theories and models in genetics, ecology, and evolution, and can be a powerful tool in combination with studies on living organisms (Adami, 2006; Kawecki et al., 2012).

## Experimental Evolution of Microbe–Microbe Mutualisms

Experimental evolution has helped answer a broad range of questions, such as elucidating the role of genetic and environmental variations in adaptation, characterizing life history and reproductive traits, and evaluating the potentials and limits of intra- and inter-species interactions (Reznick et al., 1990; de Visser et al., 1999; Morran et al., 2011; Ratcliff et al., 2012). Microbes have been the model organisms for experimental evolution due to their fast generation time, high fecundity, relatively smaller and more easily manipulated genomes, ease of laboratory rearing, and the ability to be cryogenically preserved (Elena and Lenski, 2003).

Many experimental studies have evolved mutualisms between microbes that are not known to naturally associate with one another. These beneficial behaviors can occur within and across microbial species, as well as across domains of life (as well as in bacteriophages, e.g., Sachs and Bull, 2005). A major facilitator of these mutualistic interactions involves the removal of essential nutrients from the environment or genes involved in synthesizing these nutrients (Shou et al., 2007; Harcombe, 2010; Hillesland and Stahl, 2010). For example, Hillesland and Stahl (2010) evolved an obligate mutualism between a sulfate-reducing bacterium, *Desulfovibrio vulgaris*, and an archaeon, *Methanococcus maripaludis*, in the absence of substrates that would otherwise allow them to grow independently of each other. The bacterium produces hydrogen during an energy-producing reaction, while the archaeon feeds on the hydrogen product, keeping the energy reaction going in the bacterium. This interaction allows the bacterium to produce enough energy to grow and provides the only substrate for growth that the archaeon can use. Co-cultures of evolved strains grew faster than co-cultures of the ancestral strains under similar environments, indicating a mutualism had evolved between the species after 300 generations. A subsequent study determined that several populations of the co-cultured bacteria had lost their ability to reduce sulfate, thus preventing them from proliferating without the archaeon (Hillesland et al., 2014). Such studies demonstrate that microbe–microbe mutualisms can be evolved using experimental evolution. Much less work has been done with eukaryotes.

## Experimental Evolution of Host–Pathogen Interactions

Eukaryotes have been utilized across a range of different evolutionary experiments (Garland and Rose, 2009), including in studies of antagonistic host–microbe interactions. Experimental evolution across multiple systems has provided empirical evidence of the principles and mechanisms involved in host-parasite interactions and antagonistic coevolution (Ebert and Mangin, 1997; Brockhurst et al., 2007; Morran et al., 2011; Schulte et al., 2011; Kawecki et al., 2012; Kerstes et al., 2012; Brockhurst and Koskella, 2013). For example, in an experiment between the red flour beetle and its microsporidian parasite, Kerstes et al. (2012) found that populations that coevolve with their parasite exhibited an increase in recombination rate compared to populations without parasites. Another study by Morran et al. (2011) found a higher rate of outcrossing in *Caenorhabditis elegans* populations coevolving with pathogenic *Serratia marcescens* compared to populations not exposed to parasites or where *S. marcescens* was not evolving alongside *C. elegans.* These studies demonstrate that coevolutionary interactions with parasites can lead to the maintenance of supposedly costly mechanisms in hosts (recombination and outcrossing) because they generate genetic diversity that allows hosts to combat parasites. Coevolutionary experiments also have provided evidence for host-parasite local adaptation, such that specificity evolves between host and parasite populations that coevolve together (Lohse et al., 2006; Koskella and Lively, 2007; Morran et al., 2014; Gibson et al., 2015). For example, coevolution of *Paramecium caudatum* with its bacterial parasite, *Holospora undulata*, showed that hosts are more resistant against parasites with which they coevolved, but incur a cost when the parasite is absent (Lohse et al., 2006). These studies highlight the importance of coevolutionary interactions in shaping the evolutionary trajectories of both hosts and microbes. Additionally, they show that host populations can evolve measureable phenotypic changes during experiments. In all cases, the experiments were testing specific theoretical predictions relating to host-parasite coevolution and local adaptation. Overall, these studies demonstrate the tractability and rapid evolution of eukaryotic hosts and their microbes, providing further evidence that model systems can be exploited for experimental evolution of beneficial host–microbe interactions. The success of experimental evolution in characterizing relationships between hosts and parasites is evidence that beneficial host–microbe studies would gain from using the approach as well. Despite a large body of work utilizing experimental evolution to study host-parasite interactions, there have been few experimental evolution studies examining beneficial behaviors between eukaryotic hosts and microbes.

## Utilizing Experimental Evolution to Study Beneficial Animal–Microbe Associations

### Reduction in Host–Microbe Antagonism

The first steps in the origin of a beneficial association may be a reduction in antagonism in an existing parasitic or predatory relationship (Jeon, 1972; Degnan et al., 2009). Once conditions (e.g., environmental, genetic) are met such that benefiting the host is better for the microbe and vice versa, the transition from parasitism toward mutualism may take place during a relatively short timescale. For example, Marchetti et al. (2010) evolved *Ralstonia solanacearum*, a plant pathogen, into a potential beneficial bacterial symbiont of a legume, *Mimosa pudica*. The authors inserted a plasmid containing nitrogen-fixation and nodule-forming genes into the pathogen, and allowed the plant to select for bacterial strains that can form nodules, which typically house nitrogen-fixing bacteria. The authors alternated bacterial passages within and outside the plant host, simulating bacterial movement between soil and host plant. They were able to improve the bacterium’s nodulating and infecting abilities, as well as its ability to reduce host immune responses (Marchetti et al., 2014). Furthermore, genomic manipulation of these bacterial strains suggest that error-prone mechanisms facilitated evolution toward symbiosis due to temporary increases in genetic diversity (Remigi et al., 2014). Even though the bacteria were not able to fix nitrogen (and confer benefits to the host), these experiments established the initial steps (improved infecting and nodulating capabilities) needed for a mutualistic association to evolve.

In terms of animal models, there are several examples of evolution toward a more beneficial interaction involving the model nematode, *C. elegans.* Gibson et al. (2015) found that 20 generations of coevolution of *C*. *elegans* with its parasite, *S. marcescens*, resulted in higher fecundity in hosts relative to when only the host or parasite population was permitted to evolve in the presence of the other species, leading to a reduction in antagonism in this parasitic association. Another study used *C. elegans* to explore trade-offs to host adaptation in *Burkholderia cenocepacia* (Ellis and Cooper, 2010). *B. cenocepacia* was evolved on onion medium for 1,000 generations before switching to *C. elegans*, where the bacterium exhibited reduced ability to kill the nematode. Finally, perhaps the most direct evidence of a parasitic microbe transitioning into a protective microbe is from a recent study by King et al. (2016). The authors experimentally evolved the bacterium *Enterococcus faecalis* to protect *C. elegans* against the more virulent *Staphylococcus aureus* over 15 host generations, despite the fact that these species were not known to be associated previously, thus establishing a novel host–microbe association with known evolutionary history and origin over an experimentally tractable time scale. The study also shows that evolution of bacterial protection can be rapid and can occur apart from any significant change in the host. Overall, these studies illustrate the power of experimental evolution to potentiate the transition from parasitism toward a beneficial association. Additional experiments are necessary to determine whether these interactions can be evolved to further increase the fitness of both host and microbe, as well as the stability of the interaction and how it could move toward a long-term beneficial symbiosis.

### Evolutionary Interactions between Host and Microbe

Different types of evolutionary interactions between host and microbe likely have an important role in the evolution of beneficial symbioses. Coevolution may be a driving force behind the evolution of mutualistic associations because it can create genotypes that fit well together (Guimarães et al., 2011). However, host and microbe need not be coevolving together in order for a symbiosis to evolve. The microbe may undergo evolutionary changes in the presence of the host without the host evolving itself, or vice versa (Janzen, 1980; Moran and Sloan, 2015). Several experiments tracking the evolution of microbes within non-evolving hosts have provided insight into symbiotic interactions (Sachs and Wilcox, 2006; Schuster et al., 2010; Kubinak and Potts, 2013; Barroso-Batista et al., 2014). For example, Schuster et al. (2010) passaged bioluminescent *V. fischeri* strains of fish and free-living origins through the squid, *Euprymna scolopes*, where it acted as the selective agent for a few hundred bacterial generations. The authors found that these bacteria evolved reduced bioluminescence, a trait exhibited by *V. fischeri* native to the squid host, indicating that natural selection can facilitate rapid bacterial adaptation to non-native hosts and potentially in the evolution of close symbiotic relationships.

Although there have been few evolution experiments utilizing mammalian systems to examine gut microbiota, a recent study completed a selection experiment on a non-model mammal—the bank vole—to characterize its microbial composition. Kohl et al. (2016) examined the changes in gut microbial community that occurred after selecting for voles adapted to a high-fiber, herbivorous diet. By comparing the microbiota of selected hosts against that of the control, randomly bred hosts, the authors determined that the herbivorous diet led to a more diverse microbial community. Interestingly, the individuals whose microbiota were sampled had not been exposed to the herbivorous diet themselves (they were offspring of those fed the herbivorous diet), suggesting that the differences in microbial communities were not due to the transient effects of diet and may be due to selection acting on certain microbial members. While mammalian systems are generally more difficult to maintain compared to invertebrates, this study highlights the utilization of experimental evolution of mammals to investigate complex microbial communities, which are often absent in invertebrate models. Moreover, by performing the experiment in a controlled setting, the study contributes empirical support toward the current literature, which traditionally has been mainly comparative studies, on the role the gut microbiota has on herbivore evolution.

### From Close–Knit Associations to Breakdown of Beneficial Symbioses

In many insect symbioses, the insect host harbors secondary symbionts that are part of the host’s defense mechanism. Because most of these symbionts are maternally inherited, they depend on host survival to improve their fitness (Oliver et al., 2013). This interaction presents another layer of complexity between the host, its symbiont, and enemies of the host. Because the host now possesses a more dynamic defense system, it places different selective pressures upon enemies of the host compared to innate host immunity alone. Enemies can also place selective pressure upon the symbiont and innate host immunity, leading to a three-way interaction where each species can evolve in response to the others. A recent study sought to explore this three-way interaction between *Drosophila melanogaster* innate resistance, *Drosophila* C virus, and the fly’s *Wolbachia* symbiont known to confer protection against the virus (Martinez et al., 2016). Fly populations where *Wolbachia* was either present or absent were exposed to the virus for nine generations. The authors then quantified the frequency of an allele in the flies known to confer resistance to the virus, where they found the resistant allele to be lower in frequency in populations harboring *Wolbachia* compared to those without *Wolbachia*. This experiment also provides evidence supporting the observation that hosts harboring protective symbionts tend to have a weaker immune system because they do not depend on innate immunity as much as those lacking the symbionts (Gerardo et al., 2010).

Similarly, a few studies have taken advantage of the well-developed aphid models to explore long-term symbiosis in greater depth. Dion et al. (2011) examined the evolution of the pea aphid parasitoid wasp, *A. ervi*, in the presence of the protective bacterium, *H. defensa*, which decreases survivorship of parasitoid eggs laid in the aphid. Parasitoids were exposed to clonal aphid hosts harboring or free of *H. defensa* for 10 generations, after which they were assayed for parasitism ability. The experiment showed that even though *H. defensa* reduced parasitoid offspring number in the first few generations, parasitoids eventually exhibited similar parasitism rate regardless of the presence or absence of *H. defensa*. A later study further evaluated the role of *H. defensa* by experimentally evolving the parasitoid wasp, *Lysiphlebus fabarum*, of the black bean aphid (Rouchet and Vorburger, 2014). By infecting the same aphid clone with either of three different strains of *H. defensa*, the authors directly tested parasitoid adaptation against these strains while controlling for host genotype over 11 generations. They found that increased success in parasitizing hosts harboring one symbiont strain did not lead to adaptation to the other two strains. Overall, these studies investigate the relative roles of symbiont-conferred protection vs. innate host resistance and lend further support to the idea that symbionts are an important source of variation in host defense.

Experimental evolution has also been used to identify factors that breakdown or stabilize animal–microbe partnerships. For example, Sachs and Wilcox (2006) evolved an algal symbiont of the upside-down jellyfish, which normally provides benefits to its host through the production of photosynthates, into a partner that reduced the fitness of the host by altering the mode of symbiont transmission between host generations. However, more studies would be helpful to assess whether it is difficult to breakdown associations once host and microbial interests have aligned. For example, to determine if there is a cost to maintaining a mutualistic association under some conditions (i.e., context-dependent mutualism), we can place a mutualistic host–microbe population under different environmental conditions (i.e., in the presence and absence of biotic and abiotic stressors). The host and microbial populations can then be monitored over several generations to determine if the interaction is mutualistic across all contexts, and, if not, what are the consequences for the stability of partnerships when there is environmental contingency in fluctuating environments.

### Remaining Questions and Future Directions

**FIGURE 1:**
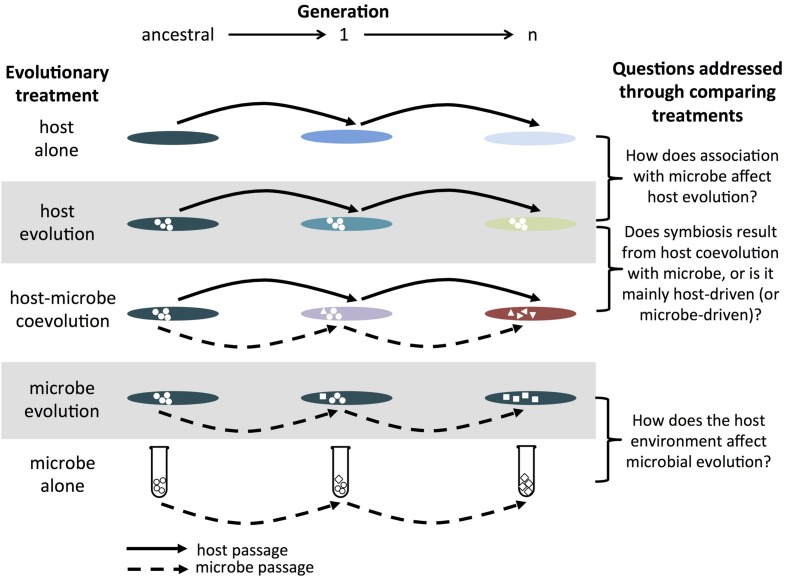
**Example of host–microbe experimental evolution experiment**. The host alone treatment consists of passaging the host without the microbe (and vice versa for the microbe alone treatment). The host evolution treatment consists of passaging the host in the presence of a non-evolving microbe (and vice versa for the microbe evolution treatment). The host–microbe coevolution treatment consists of passaging hosts and microbes that have interacted with each other in the previous generation. In combination, these treatments allow researchers of symbioses to assess the effects of partner association and coevolution on the evolution of hosts and microbes. While a single replicate of each treatment is shown for simplicity, replicate populations within each treatment are critical in order to evaluate the relative roles of deterministic and stochastic processes. The greater the contribution from stochastic processes, the greater the degree of divergence between replicate populations. Note, this is a similar set-up as that proposed for studying host-parasite coevolution in Brockhurst and Koskella (2013).

While the previous sections have provided several examples of how experimental evolution has been used to study host–microbe associations, more work is needed to create a more thorough understanding of the evolution of beneficial animal–microbe symbioses. Prior studies have set the stage for much wider investigations into the proximate and ultimate mechanisms shaping the evolution of these associations. For example, evolving microbes that have had little contact with eukaryotic hosts into host-associated microbes could elucidate the mechanisms and consequences resulting from the microbial transition from free-living to endosymbiosis. Sequencing the genomes of ancestral and evolved populations would then provide insight into the initial genomic modifications important for a transition toward adaptation to a host. Likewise, by performing one-sided evolution experiments alongside coevolution experiments, we can identify the traits that have arisen as a result of the presence of a partner or through selection that hosts and microbes impose on each other (an example of such a setup is shown in **Figure 1**). Indeed, studies of coevolution of *de novo* mutualism between a eukaryotic host and its microbes should be considered an important next step in symbiosis research. Experimental evolution can also be used to understand why some symbioses are difficult to breakdown and the mechanisms involved in maintaining these relationships (Morran et al., 2016). Lastly, there have been few evolutionary studies examining the dynamics between host immunity and beneficial/protective microbes and how they influence microbial and host evolution. To test the importance of host defenses in the initial stages of a beneficial association (Doebeli and Knowlton, 1998), we can evolve hosts differing in immune responses with the same microbial genotype to determine the evolutionary trajectories taken by each host–microbe pairing. Comparison of the immune responses of the ancestral and evolved hosts would provide further insight into the extent that microbes can alter host defenses over time (Kitano and Oda, 2006).

In general, because evolution experiments control and manipulate environmental conditions and starting population genetics, we can use them to test hypotheses and predictions that have been proposed for the evolution of beneficial association. By using mutants or by manipulating the environment (such as by removing important dietary substances or imposing selective pressures from an enemy), then measuring the fitness of both hosts and symbionts, we can identify the biotic and abiotic factors that influence the establishment and maintenance of mutualisms. Finally, through replicate populations, we can determine whether deterministic forces (e.g., selection) are more dominant in the evolution of beneficial symbioses compared to stochastic forces (e.g., mutation and genetic drift). Below, we suggest a few animal models for use in evolutionary experiments of beneficial associations.

### Animal Systems for Experimental Evolution of Beneficial Interactions

The ideal model system for experimental evolution of beneficial host–microbe interactions would include several aspects. For the host, a short generation time and easy laboratory maintenance would allow for replicate experiments and observable host evolution. For the microbe, the ability to be cultivated outside of the host would be advantageous for examining the evolution of the microbe in the presence and absence of the host. Additional traits for both hosts and microbes, such as availability of genetic tools and genomic resources, small genomes, and cryogenic storage, would help to link specific genotypes to observed phenotypes. Of course, not every one of these conditions must be met in order to address some questions. For example, even when host populations cannot be studied for a large number of generations, evolving the microbial population within hosts may lend insight into host–microbe dynamics (Sachs and Wilcox, 2006; Schuster et al., 2010; Kubinak and Potts, 2013; Barroso-Batista et al., 2014).

**FIGURE 2:**
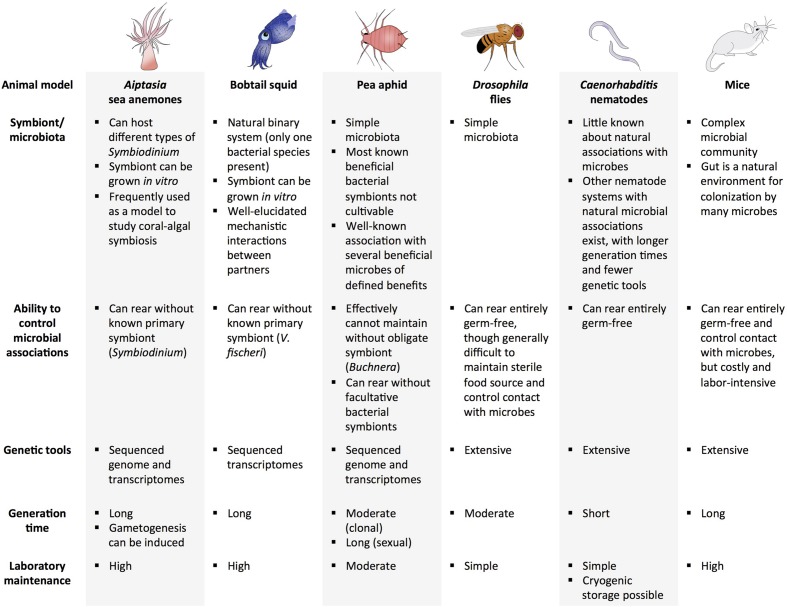
**Examples of potential animal model systems for experimental evolution of beneficial host–microbe interactions**.

Several animal model systems have potential to be used for experimental evolution of beneficial host–microbe interactions (a subset of systems are highlighted in **Figure 2**). The ubiquity of marine symbioses has made cnidarian-protist associations some of the most widely studied systems in symbiosis research. A challenge of marine systems has been a lack of tractability of the host and inability to culture the symbiont without the host. Additionally, although protists make up a large proportion of described beneficial microbial associations with marine invertebrates, they have relatively large genomes, making sequencing more difficult than other types of symbionts. However, advances in technology have facilitated genomic and transcriptomic analyses of several cnidarians and their symbionts (Shinzato et al., 2011; Bayer et al., 2012; Lehnert et al., 2012; Artamonova and Mushegian, 2013; Baumgarten et al., 2015). For example, an emerging model for symbiosis establishment is the sea anemone *Aiptasia*, which forms an association with *Symbiodinium*, the algal symbiont of many cnidarians, including corals. Due to its relative ease of laboratory rearing, ability to be maintained without a symbiont, and sequenced genome and transcriptomes, *Aiptasia* is a highly tractable model for studying cnidarian-protist interactions (Lehnert et al., 2012; Baumgarten et al., 2015). Furthermore, induction of spawning is possible in the laboratory, where abundant larvae can be produced when needed (Grawunder et al., 2015).

Another marine animal that has been studied extensively to investigate beneficial animal–microbe associations is the Hawaiian bobtail squid, *E. scolopes*. It harbors only one type of bacterium in its light organ—*V. fischeri—*which it obtains from the environment. The ability to grow the bacterium separately from its host is advantageous because hosts and bacteria can be evolved independently and together. Research on this system has also provided essential findings on the mechanisms involved in partner identification and communication (Davidson et al., 2004; Nyholm and McFall-Ngai, 2004; Kremer et al., 2013). Other advantages and considerations for use of the squid-*Vibrio* system in experimental evolution are reviewed in Soto and Nishiguchi (2014). Limitations of this system include the generation time of the squid host (first eggs are laid around 60 days post-hatching in the laboratory), and relatively high maintenance of proper environmental conditions (e.g., water quality, lighting) and food sources (Hanlon et al., 1997), which may hamper studying host evolution.

Insects and their symbionts have been widely used models for symbiosis due to their tractability and relatively simple association with microbes (only a few microbes are present in some insect hosts). Among insect-microbe symbioses, the pea aphid and its bacterial symbionts are among the best characterized. Aphids harbor several beneficial symbionts, including *Buchnera*, an obligate intracellular bacterium that exchanges amino acids with its host. Genomic aspects of host and *Buchnera* interaction are well-defined (Wilson et al., 2010). Aphids also harbor several other symbionts that provide protection against natural enemies (Scarborough et al., 2005; Vorburger et al., 2010; Parker et al., 2013). The aphid-symbiont system is an excellent model for evaluating context-dependent factors involved in beneficial interactions, as well as the three-way interaction between innate host defense, protective symbionts, and natural enemies of the host (Dion et al., 2011; Weldon et al., 2013; Polin et al., 2014; Rouchet and Vorburger, 2014). Although most bacterial symbionts in aphids cannot be grown *in vitro* (an exception is found in Renoz et al., 2015), it is possible to replace natural *Buchnera* strains with *Buchnera* from another aphid lineage or facultative bacterial strains, thus opening the possibility for further study of these tightly knit associations (Koga et al., 2003; Moran and Yun, 2015). One challenge of studying host evolution in this system is that even though aphids can reproduce sexually and asexually, it is only practical to propagate aphids clonally in the lab, resulting in little genetic variation over experimentally relevant timescales. Although sexual reproduction would increase genetic variation through recombination, this mode of reproduction would take much longer than clonal reproduction.

Other insect systems have been utilized in beneficial host–microbe experiments and experimental evolution studies. Research on the microbiota of *Drosophila* has highlighted the role microbes have in host development and protection from natural enemies, paving the way for studying the influence of a microbial community on host evolution (Storelli et al., 2011; Hamilton et al., 2015; Mateos et al., 2016). The fruit fly has also been used in a wide range of evolution studies, from research on temperature adaptation to learning abilities (Dunlap and Stephens, 2014; Schou et al., 2014), and in experiments as long as 600 generations (Burke et al., 2010). Additional advantages include sequenced genomes and gene manipulation tools, and elucidated pathways involved in microbe-mediated host development, immune response, behavior, and intestinal activities (Kuraishi et al., 2013; Lee and Brey, 2013). Germ-free organisms can be established to facilitate comparison of host evolution in the presence and absence of microbes; however, it is relatively difficult to maintain a sterile food source and control contact with microbes in *Drosophila*. Similar to *Drosophila*, there is also growing interest in the microbiota of mosquitoes, particularly its influence on human pathogen transmission (Jupatanakul et al., 2014; Hegde et al., 2015). Although a few evolution experiments have been conducted with and within mosquitoes (Yan et al., 1997; Vasilakis et al., 2009; Legros and Koella, 2010), there are many novel approaches in which mosquitoes and their microbes could be exploited to further insight into how host and microbes adapt to one another, and, importantly, how this might influence vectorial capacity of important disease vectors.

*Caenorhabditis elegans* is an invertebrate system that has been utilized in numerous experimental evolution studies (Gray and Cutter, 2014). Like *D. melanogaster*, *C. elegans* has many genetic tools available and has a very short generation time for a eukaryote. Although not much is known about its natural associations with microbes, the nematode has been used extensively as a model for studying evolution of host-parasite interactions and microbe-mediated immune responses (Couillault and Ewbank, 2002; Irazoqui et al., 2010; Dunbar et al., 2012; Portal-Celhay and Blaser, 2012; Lee et al., 2013; Ermolaeva and Schumacher, 2014). Other nematode systems exhibiting long-standing mutualisms with microbes also exist (Goodrich-Blair, 2007; Murfin et al., 2012; Clarke, 2014). For example, the symbiotic interaction between the nematode, *Steinernema carpocapsae*, and its bacterial symbiont, *Xenorhabdus nematophila*, is well characterized (Cowles et al., 2007; Cowles and Goodrich-Blair, 2008; Chaston et al., 2013), and the system has been utilized for several experimental evolution studies (Bashey et al., 2007; Vigneux et al., 2008; Bashey and Lively, 2009; Chapuis et al., 2012; Morran et al., 2016). However, the system is generally less amenable to experimental evolution relative to *C. elegans*, because *S. carpocapsae* has a longer generation time and fewer available genetic tools. Nonetheless, the *S. carpocapsae* system can be an effective tool for testing hypotheses regarding established mutualisms.

Vertebrates are seldom recognized as models for experimental evolution, partly because they are relatively more difficult to maintain in the laboratory (e.g., expenses and animal care regulations) and have a long generation time. However, the microbiota of vertebrates, particularly mammals, are often much more complex than invertebrates, and thus provide a compelling model to examine host–microbiome evolutionary dynamics. The presence of the adaptive immune system in vertebrates also allows for further elucidation of the interactions between the immune system and the microbiome (Kitano and Oda, 2006). Some vertebrate models, including mice and zebrafish, can be reared to be germ-free until introduction of microbial communities (Ruby, 2008). Several experimental evolution studies have utilized mice as a model, such as artificial selection studies of nest-building and wheel running, and natural selection experiments of mating systems and captivity (Garland and Rose, 2009; Lacy et al., 2013; Firman et al., 2015). Microbial evolution experiments have been done in mouse models, where microbes are allowed to evolve within the host, providing a better look at how the host environment (e.g., host immunity) and microbial community can affect microbial adaptation (Kubinak and Potts, 2013; Barroso-Batista et al., 2014). Native and introduced microbial communities have also been extensively examined in mice models (Hasegawa and Inohara, 2014; Laukens et al., 2015). Similarly, zebrafish has great potential for use in host–microbe evolution studies, particularly those addressing alteration of host immune responses by members of the microbial community (Kanther et al., 2011; Rolig et al., 2015).

While no single animal system is ideal in answering every outstanding question in symbiosis research, these highlighted systems are poised to address many of the present questions in beneficial symbioses. By using classic model organisms with fast generation times, high fecundity, and low maintenance (such as *Drosophila* flies and *Caenorhabditis* nematodes), we can determine the fundamental steps necessary for the evolution of mutualism between a eukaryotic host and a microbe. Performing experimental evolution with established symbiotic systems (such as the pea aphid and bobtail squid) can elucidate mechanisms involved in maintaining particular associations and may allow us to retrace the pathways leading to these evolutionarily stable associations. While vertebrate-microbe systems may be more difficult to establish, the success of previous experiments with single rodent species (Garland and Rose, 2009; Lacy et al., 2013; Firman et al., 2015) and microbial evolution within mammalian hosts (Kubinak and Potts, 2013; Barroso-Batista et al., 2014) has shown that host–microbe coevolution experiments with vertebrates is possible.

## Conclusion

Our understanding of beneficial host–microbe interactions is based largely on studying established associations. More direct tests are needed to solidify our understanding of how hosts and microbes interact with and affect each other. Experimental evolution, which has succeeded in bridging theory and empirical tests of fundamental evolutionary processes, can provide a way to examine these interactions, particularly to test those predictions involving the evolution of host–microbe mutualisms. Current model systems in both microbial symbiosis and experimental evolution studies are poised for further explorations of beneficial interactions between animal hosts and their microbes. With more studies of symbioses utilizing experimental evolution, we can then further our understanding of the mechanisms involved in the establishment, maintenance, and short- and long-term consequences of beneficial host–microbe associations for both hosts and microbes.

## Author Contributions

KH, LM, and NG developed the ideas presented here. KH wrote the manuscript, with revisions and edits from LM and NG.

## Conflict of Interest Statement

The authors declare that the research was conducted in the absence of any commercial or financial relationships that could be construed as a potential conflict of interest.
